# Designing Biodegradable Wafers Based on Poly(L-lactide-co-glycolide) and Poly(glycolide-co-ε-caprolactone) for the Prolonged and Local Release of Idarubicin for the Therapy of Glioblastoma Multiforme

**DOI:** 10.1007/s11095-020-02810-2

**Published:** 2020-05-07

**Authors:** Artur Turek, Katarzyna Stoklosa, Aleksandra Borecka, Monika Paul-Samojedny, Bożena Kaczmarczyk, Andrzej Marcinkowski, Janusz Kasperczyk

**Affiliations:** 1 Faculty of Pharmaceutical Sciences in Sosnowiec, Medical University of Silesia, Katowice, Chair and Department of Biopharmacy, Jedności 8, 41-200 Katowice, Poland; 2 Centre of Polymer and Carbon Materials, Polish Academy of Sciences, M. Curie-Sklodowskiej 43, 41-819 Zabrze, Poland; 3 Faculty of Pharmaceutical Sciences in Sosnowiec, Medical University of Silesia, Katowice, Chair and Department of Medical Genetics, Jedności 8, 41-200 Sosnowiec, Poland

**Keywords:** glioblastoma multiforme, idarubicin, poly(glycolide-co-ε-caprolactone), poly(L-lactide-co-glycolide), wafer

## Abstract

**Purpose:**

The blood-brain barrier limits the application of idarubicin in the therapy of glioblastoma multiforme. Biodegradable, intracranial wafers with prolonged release may increase therapy efficiency.

**Methods:**

Blank wafers, wafers containing 5% *w*/w and 10% w/w of idarubicin were formulated by solution casting from poly(L-lactide-co-glycolide) and poly(glycolide-co-ε-caprolactone). The following methods were used: NMR, GPC, DSC, FTIR, AFM, UV-VIS, and a viability and proliferation assay for idarubicin action (U87MG cell line).

**Results:**

Wafers showed a surface with numerous immersions and hills. A lack of interactions between idarubicin and the copolymers was observed. The substance was entrapped in the matrix and released in two phases for all wafers with the appropriate bolus and maintenance dose. The burst effect was observed for all wafers, however, the biggest bolus for poly(L-lactide-co-glycolide) wafers containing 5% w/w of idarubicin was noted. The stable and steady degradation of poly(glycolide-co-ε-caprolactone) wafers containing 5% w/w of idarubicin ensures the most optimal release profile and high inhibition of proliferation.

**Conclusions:**

Copolymer wafers with idarubicin are an interesting proposition with great potential for the local treatment of glioblastoma multiforme. The release rate and dose may be regulated by the amount and kind of wafers for various effects.

## Introduction

Idarubicin (IDA) belongs to the group of anthracycline antibiotics and turns out to be effective against leukemia and sarcoma at lower doses than daunorubicin. In the last two decades, IDA was routinely used *inter alia* in the treatment of acute myeloid leukemia, lymphoblastic and nonlymphoblastic leukemia, and also in metastatic breast cancer. IDA is also suitable for use in other diseases because it inhibits nucleic acid synthesis and the interaction with the enzyme topoisomerase II. An *in vitro* study on the inhibition of the growth of glioblastoma multiforme (GBM) rat cells by IDA and its metabolite idarubicinol has revealed that this drug substance should be appropriated for the treatment of malignant brain tumor tissues (1,2). Moreover, the high lipophilicity of IDA through the lack of a methoxy group at position 4 of the anthracycline predisposes it to optimal penetration into brain tissue in comparison to other anthracyclines (3). However, clinical trials on this issue showed a significant limitation in IDA efficiency in the treatment of brain tumors. According to a phase II clinical trial, IDA administered by intravenous injection in pediatric patients with relapsed brain tumors was not sufficiently active against medulloblastoma, ependymoma or brain stem tumors (4). Dreyer and co-workers (2003), based on a phase II clinical trial of IDA in pediatric brain tumors, point out that infusions were not efficient because of the limited uptake through the blood-brain barrier (5). A higher IDA concentration in brain tumors may be achieved by increasing the dose, however, this is connected with side effects. Analysis of the literature data revealed significant side effects as a result of oral or intravenous administration of IDA, e.g. consistent myelosuppression, gastrointestinal, dermatologic, cardiologic, hepatic or renal side effects.

Nowadays, IDA is commercially available only as a powder of IDA hydrochloride for use in an aqueous solution for intravenous injections and oral hard gelatin capsules. The optimization of methods for the therapy of GBM, limiting adverse reactions and the problem of side effects may be solved by locally administrated intracranial biodegradable formulations.

According to the literature data, conjugates (6), nanoparticles (7), micelles, liposomes (8–11), microspheres (12) and beads (13) were developed to modify the release profile of IDA. The mentioned formulations are based on derivatives of polypeptides (6), polysaccharides (12), lipids (7), alcohols (8–10) and polyesters. These formulations have limitations in durability in aqueous media and in the amounts for drug loading. For the listed formulations, the release period does not exceed a week (6–12). None of these formulations are dedicated to GBM treatment and cannot be adopted for intracranial injection. However, some solutions for GBM based on various drug substances and drug carriers were proposed (e.g. chitosan engineered polyamidoamine dendrimers with temozolomide or lomustine nanoparticles), but they concentrate on uptake through the blood-brain barrier. These formulations also have the limitations in their stability, the amount of drug incorporation and prolonged release (14,15).

It should be pointed out that GBM is a fatal primary brain tumor with a median survival of ⁓18 months (16). GBM is characterized by a rapid invasion and relapse. Therefore, effective therapy should provide a bolus dose and maintenance release over 18 months. Nowadays, none of the proposed formulations with IDA meet these requirements.

The routine methods of GBM treatment include maximal safe resection of the tumor, partial brain radiotherapy and oral or intravenous chemotherapy (16). A more effective solution is combination therapy of maximal safe resection, partial radiotherapy and local chemotherapy by implantable drug formulation. However, the last component of therapy plays a key role.

Recently, some studies on intracranial formulations were performed. Poly(glycolide-co-lactide) wafers containing fenofibrate combined with standard chemotherapy or metronomic treatment may limit both the danger of recurrence and reduce severe toxicity and as a consequence lead to a substantial improvement in the prognosis for patients (17). A biodegradable hybrid-structured nanofibrous membrane with O^6^-alkylguanine, carmustine and temozolomide is also proposed to enhance therapeutic efficacy (18). However, the last animal studies of various chemotherapeutics (carboplatin with carmustine, 5-fluorouracil, paclitaxel) and last clinical study for 5-fluorouracil encapsulated in polymeric microspheres for localized glioma therapy by intracranial administration demonstrated significant potential to bring the therapy to early stage clinical evaluation (19).

Nowadays, the Gliadel® wafer is one biodegradable and intracranial medicinal product, containing carmustine as the drug substance and based on a polyanhydride copolymer of poly[bis(*p*-carboxyphenoxy) propane] and sebacic acid (80:20). It was evidenced that ∼60% of the carmustine was released from the wafer into the brain tissue over three days in a rabbit model (20). According to the last meta-analysis of the role of Gliadel® wafers in the treatment of newly diagnosed GBM (six studies including two randomized controlled trials and four cohort studies for 513 patients) performed by Xing and co-workers, intracranial Gliadel® wafers are significant in improving survival (21).

The solutions proposed in this work are based on wafers formulated from aliphatic polyesters providing the release of IDA after tumor resection with a bolus dose and a maintenance dose over the relapse period.

From a therapeutic point of view, IDA possesses more advantages than carmustine. The study of Lünenbürger and co-workers on the antiproliferative effects on BT16 cell lines from a human brain tumor revealed that the dose required to inhibit growth by 50% after 72 h of incubation was 80 times lower for IDA compared to carmustine (22).

In this study, wafers with IDA were formulated from poly(L-lactide-co-glycolide) (L-PLGA) or poly(glycolide-co-ε-caprolactone) (PGACL). Both aliphatic polyesters were synthetized with a low toxic initiator (zirconium (IV) acetylacetonate) (23,24).

The aim of our work was to design biodegradable intracranial wafers based on L-PLGA and PGACl for the prolonged and local release of idarubicin for the treament of GBM. The following aspects have been developed for the wafer design: (i) formulation of wafers by the solution casting method, (ii) determination of the release profiles, (iii) surface properties, and (iv) the composition and chain microstructure during degradation for selected wafers.

## Materials and Methods

### Copolymer Synthesis

L-PLGA 85:15 and PGACL 10:90 were synthesized at the Centre of Polymer and Carbon Materials of the Polish Academy of Sciences in Zabrze in bulk with the use of zirconium (IV) acetylacetonate as a low toxic initiator. The synthesis of copolymers was carried out using a reactor from the Parr Instrument Company (4550 Floor Stand Pressure Reactor) with computer control of the polymerization parameters. The polymerization process was conducted in a melt at 130°C for 24 h and then at 115°C for 72 h. The copolymers were purified by dissolution in chloroform and added dropwise to cold methanol. The polymer materials were then dried under vacuum conditions at 25°C. All of the monomers except ε-caprolactone (CL) were purified by recrystallization from ethyl acetate, dried in air conditions, and then dried in a vacuum oven at room temperature. The CL was dried and purified by distillation over calcium (23,25).

### Wafer Formulation

Blank wafers, wafers with 5% w/w of IDA (wafers-IDA5) (IDA hydrochloride, Pharmaceutical Research Institute, Warsaw, Poland) and with 10% w/w of IDA (wafers-IDA10) were formulated by the solution casting method at 25°C.

Before the process, the raw copolymers were dried under air conditions in a laminar box for seven days. The dry copolymers were subjected to grinding at a temperature of −196°C in a cryogenic mill (6870 SPEX, USA) and dried again with the use of a drying set containing a dryer (Memmert VO500) and a pump (BUCHI V-710) for 14 days at a temperature of 23°C and at a pressure of 80 mbar.

The drug substance and copolymers were dissolved in various solvents, i.e. IDA in 1,1,1,3,3,3-hexafluoro-2-propanol (Sigma-Aldrich, Poznan, PL) and L-PLGA or PGACL in methylene chloride (POCH, Gliwice, PL). Mixtures were deprived of air in a vacuum line and cast on a Teflon mold (formulated by an injection mold) and left for solvent evaporation in a laminar box (seven days) followed by drying in a vacuum (seven days).

For formulation of the wafers the following amounts of substrates were used: (i) wafer-IDA5 1.5 mg of IDA and 28.5 mg of polymer, (ii) wafer-IDA10 3.0 mg of IDA and 27.0 mg of polymer, (iii) 0.1 mL of 1,1,1,3,3,3-hexafluoro-2-propanol and (iv) 0.2 mL of methylene chloride.

Blank wafers were formulated in the same way. In these, IDA was not used.

The formulation method allowed wafers of 1.2 mm ± 0.015 mm in diameter and 0.2 mm ± 0.017 mm in thickness to be obtained. The measurements for the size of the wafers were evaluated by Digital Micrometer (Digmatic ABSOLUTE QuickMike 0-15 mm 227–201, MITUTOYO).

### Copolymer Composition and Chain Microstructure Study

The measurement was done by the proton nuclear magnetic resonance method (^1^H NMR) using a Bruker-Avance II Ultra Shield Plus spectrometer operating at 600 MHz (Bruker, USA) in a 5-mm tube. Dimethylsulfoxide-d6 (DMSO-d6) (POCH, Gliwice, PL) was used as a solvent. ^1^H NMR spectra were obtained at a temperature of 80°C with 32 scans, 3.74 s acquisition time and 7 μs pulse width. Tetramethylsilane was used as an internal standard. Signals observed in the ^1^H spectra were assigned to the appropriate sequences in the polymer chain according to a previously described procedure (24,26). The molar percentages of lactidyl (*F*_*LL*_), glycolidyl (*F*_*GG*_), and ε-caproyl (*F*_*CL*_) units, the average length of the lactidyl (*l*_*LL*_), glycolidyl (*l*_*GG*_) and caproyl (*l*_*CL*_) blocks in the copolymer chain as well as the degree of randomization (*R*) were calculated according to the literature noted above.

NMR analyses were performed for the raw copolymers and PGACL wafers-IDA5.

### Molecular Weight Study

Both the *M*_*n*_ and molecular weight distribution (*D*) of the raw copolymers were analyzed by gel permeation chromatography (GPC) with a Physics SP 8800 chromatograph. Tetrahydrofuran was used as the eluent with a flow rate of 1 mL/min, and Styragel columns and a Shodex SE 61 detector were used. The *M*_*n*_ was calibrated with polystyrene standards.

### Thermal Study

The thermal characteristics of the raw copolymers were analyzed by means of a differential scanning calorimetry (DSC) method (TA DSC 2010 apparatus, TA Instruments, New Castle, DE) in a range from −30°C to 220°C in a nitrogen atmosphere (flow rate = 50 mL/min). The instrument was calibrated with high purity indium. Two heating runs were performed for all samples. The first run for the initial samples and the second run for the amorphous samples were obtained by quenching from a melt (220°C). The value of the melting temperature (*T*_*m*_) was determined from the first heating run, whereas the glass transition temperature (*T*_*g*_) was determined from the second heating run as the midpoint of the heat capacity change of the amorphous sample.

### IDA-Copolymer Interaction Study

Fourier transform infrared spectroscopy (FTIR) was used to investigate the interactions of the IDA-copolymers in the L-PLGA wafers-IDA10 and PGACL wafers-IDA10. Infrared measurements were obtained by a DIGILAB FTS-40A Fourier transform infrared spectrometer (Bio-Rad, USA) in the range of 4000–400 cm^−1^ at a resolution of 2 cm^−1^ and for an accumulated 32 scans.

The copolymers used were in the form of foils obtained by their dissolution in methylene chloride and evaporating onto potassium bromide windows. The study of pure IDA was performed for an IDA pellet formulated from potassium bromide and also on its chloroform solution.

### Morphology Study

The morphology of the L-PLGA and PGACL wafers, L-PLGA and PGACL wafers-IDA5 and wafers-IDA10 were studied by atomic force microscopy (AFM) using a MultiMode (di-Veeco, USA, CA) with NanoScope 3D and working under atmospheric conditions in the tapping mode with standard 125 μm single-crystal silicon cantilevers (Model TESP; di-Veeco, USA, CA). The piezoelectric scanner had a nominal scan range 10 × 10 μm.

### Degradation Study of Wafer

The PGACL wafers-IDA5 were incubated in artificial cerebrospinal fluid (aCFS) (at a ratio of 15 mg of wafer to 1 mL of aCFS) (Alzet, USA) under shaking conditions (240 rpm) at a temperature of 37°C.

The wafers used in the degradation study were collected before degradation and every two weeks thereafter until the 126th day of degradation and then after the 1255th day of degradation. Before measurements, the matrices were air dried at room temperature in the laminar box and then under reduced pressure.

### IDA Release Study

The wafers-IDA5 and wafers-IDA10 were incubated in artificial cerebrospinal fluid (aCFS) (at a ratio of 15 mg of wafer to 1 mL of aCFS) (Alzet, USA) under shaking conditions (240 rpm) at a temperature of 37°C. The amount of IDA released in the supernatants obtained during the degradation process was estimated by UV-VIS spectroscopy. The supernatants were collected after 4 h and then every week until the 602nd day.

Optical absorption measurements of the solutions were performed at room temperature on a V-570 double-beam UV-Visible/NIR spectrometer (Jasco Analytical Instruments, USA). A deuterium lamp was used as a source of ultraviolet light and a halogen lamp was used for the visible and near-infrared parts of the light spectrum. The absorption spectra of IDA were recorded with the use of quartz cells within a spectral range from 450 to 500 nm, where the absorption bands were observed. The main absorption band of IDA was at 485 nm; a calibration curve was prepared for this band and the calibration equation was estimated.

### IDA Release Kinetics

The mechanism of IDA release was analyzed on the basis of the cumulative release percentages of IDA from the wafers *versus* time. Profiles were matched to different mathematical models: zero-order, first-order, Higuchi and Kosmeyer-Peppas (27–29).

### Viability and Proliferation Assay

The U87MG cell line (Sigma-Aldrich, Poznan, PL) from a human brain (glioblastoma astrocytoma) was cultured in a modified Eagle’s Minimum Essential Medium (ATCC, USA) supplemented with heat-inactivated 10% fetal bovine serum (ATCC) and 10 μg/mL gentamicin (Invitrogen, USA). The cell line was maintained at 37°C in a humidified atmosphere of 5% CO_2_ in air.

U87MG GBM cells were seeded in 6-well plates (at a density of 1.6 × 10^4^ cells per well), cultured overnight (24 h) and treated for 24 h with IDA at 0.22 μmol, 1 μmol, 1.6 μmol, 2.4 μmol and 3 μmol, which reflect the cumulative amounts released from PGACL wafers-IDA5 over 4 h, 7, 14, 30 and 60 days, respectively.

The effects of IDA on U87MG cell viability were quantified using a trypan blue exclusion assay. Harvested cells were mixed with an equal volume of trypan blue (10 μL) and after trypan blue staining, cell viability was determined by counting the number of cells using a Bürker chamber. The U87MG cell viability was calculated as the percentage of live cells in the total cell population.

Data were presented as mean ± SD. A one-way ANOVA and a post hoc Tukey’s multiple comparison test were used for comparing the analyzed groups. The power of all tests was not less than 1-β = 0.8. Data were analyzed with Statistica software version 10.0 (StatSoft, Inc. 2008). All of the tests were two-sided and p < 0.05 was considered to be statistically significant.

## Results

### Characterization of Raw Copolymers

The L-PLGA copolymer possessed *F*_*LL*_ 85 mol% and *F*_*GG*_ 15 mol%, whereas the PGACL copolymer contained *F*_*GG*_ 10 mol% and 90 mol% of *F*_*CL*_. Differences were observed in the chain microstructure; the *l*_*GG*_ for L-PLGA was longer (1.4) than for PGACL (0.5). Moreover, the *l*_*LL*_ was 7.0 and the *l*_*CL*_ was 5.4 for L-PLGA and PGACL, respectively. L-PLGA was semi-blocky (R = 0.5), whereas PGACL was random (R = 1.05). The coefficient *R* is a measure of the degree of randomness of copolymer chain structure and attains a value of 0 for a diblock copolymer and 1 for a completely random distribution of copolymer chain units, therefore the results indicated that the L-PLGA was semi-block (R = 0.5), whereas PGACL was random (R = 1.05) (Table I) (30).

**Table I:** Parameters Characterizing the Raw Powders of L-PLGA and PGACL

Polymer	*F*_*LL*_ (mol %)	*F*_*GG*_ (mol %)	*F*_*CL*_ (mol %)	*l*_*LL*_	*l*_*GG*_	*l*_*CL*_	*R*	*M*_*n*_ (kDa)	*D*	*T*_*m*_ (°C)	*T*_*g*_ (°C)
L-PLGA	85	15	–	7.0	1.4	–	0.50	119	2.1	158	58
PGACL	–	10	90	–	0.5	5.4	1.05	91	2.1	54	−60

*F*_*LL*_, *F*_*GG*_, *F*_*CL*_ - molar percentage of lactidyl, glycolidyl and ε-caproyl units in the copolymer, respectively; *l*_*LL*_, *l*_*GG*_, *l*_*CL*_ – average length of lactidyl, glycolidyl and ε-caproyl blocks, respectively; *R* – randomization ratio; *M*_*n*_ - number average molecular weight; *D* – molecular weight distribution; *T*_*m*_ – melting temperature; *T*_*g*_ – glass transition temperature

The *M*_*n*_ for the raw copolymers showed generally high values, i.e. 119 kDa and 91 kDa for L-PLGA and PGACL, respectively. The first DSC heating run revealed melting endotherms at 158°C and 54°C, and the second heating run revealed *T*_*g*_ at 58°C and − 60°C for L-PLGA and PGACL, respectively (Table I).

### L-PLGA-IDA and PGACL-IDA Interactions

FTIR analysis did not reveal interactions between IDA and the L-PLGA or PGACL copolymers. The results were obtained for wafers without IDA and for wafers containing 10% w/w of IDA.

**Fig. 1:**
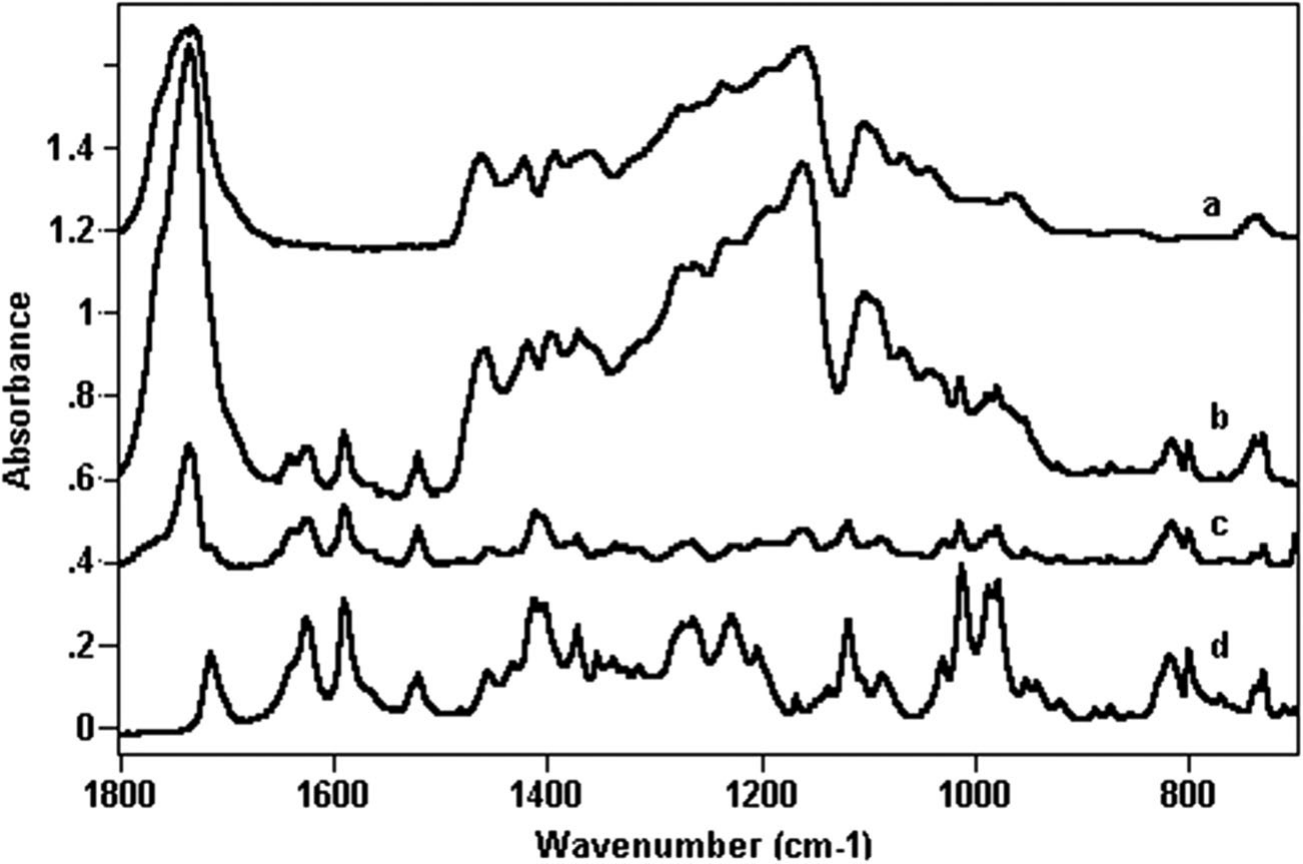
FTIR spectra in the region 1800–700 cm^−1^ of PGACL wafers (**a**), PGACL wafers-IDA10 (**b**), spectrum after subtraction of b-a (**c**), and IDA in potassium bromide pellets (**d**).

Subtracting the L-PLGA or PGACL spectra of the L-PLGA wafers-IDA10 or the PGACL wafers-IDA10 spectra revealed almost the same results as recorded for pure IDA. Figure 1 shows exemplary spectra in the region 1800–700 cm^−1^ obtained for PGACL wafers-IDA10 and blank wafers.

**Fig. 2:**
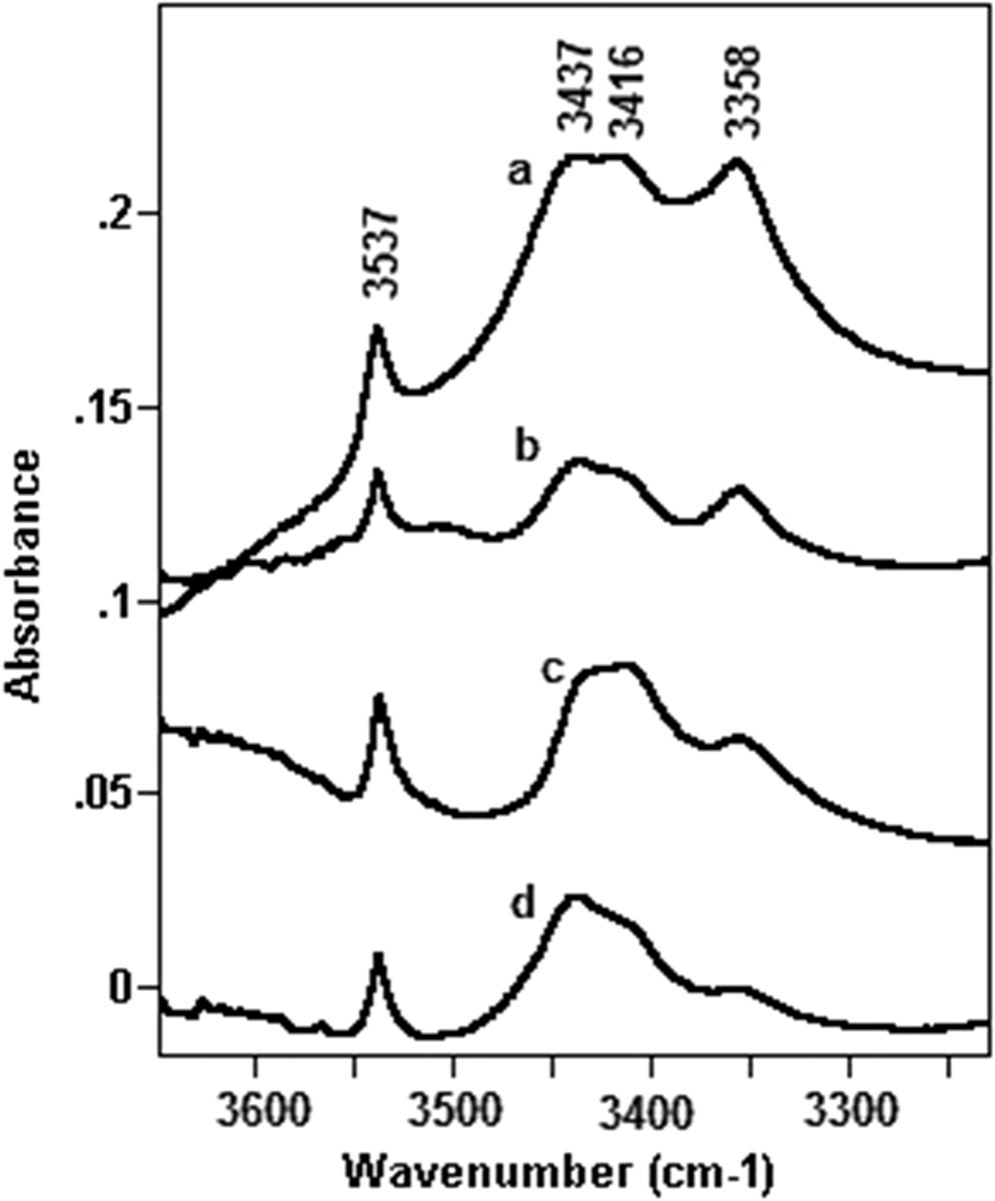
FTIR spectra in the region 3650–3230 cm^−1^ for IDA in potassium bromide pellets (**a**), spectrum of diluted IDA in chloroform (**b**), spectrum after subtraction of the spectrum of PGACL wafers (**c**), and spectrum after subtraction of the spectrum of L-PLGA wafers (**d**).

However, some small changes between the blank wafers and wafers-IDA10 were observed in the region characteristic for hydroxyl and amine groups stretching vibrations, i.e. in the range of 3650–3230 cm^−1^ (Fig. 2). In the case of the spectrum of pure IDA, four bands at 3537, 3437, 3416 and 3358 cm^−1^ appeared (Fig. 2a). In an aim to assign observed bands to a particular type of hydrogen bonds, the spectrum of diluted IDA in chloroform was recorded (Fig. 2b). The dilution only caused the breaking of intermolecular bonds with simultaneous forming of free hydroxyl groups. It appeared that the band at 3358 cm^−1^ was diminished, while the intensity of the band at 3537 cm^−1^ slightly increased. The other two bands did not change after dilution. Consequently, the band at 3358 cm^−1^ corresponded to intermolecular bonds while the bands at 3416 and 3437 cm^−1^ indicated the forming of intramolecular ones. Additionally, the infrared spectra proved that in the pure IDA sample mostly intramolecular hydrogen bonds were formed.

The introduction of IDA to the L-PLGA wafer caused the band at 3358 cm^−1^ to be slightly diminished and simultaneously the intensity of the band at 3537 cm^−1^ slightly increased (Fig. 2d). The same effect was observed in the case of sample dilution (Fig. 2b). The lack of new bands in the spectrum for L-PLGA wafer-IDA10 did not indicate the forming of hydrogen bonds between IDA and L-PLGA. In the case of the spectrum obtained by subtracting the PGACL spectrum from that for IDA-PGACL, almost the same spectrum for solid IDA in the form of a pellet formulated from potassium bromide was observed (Fig. 2c).

### Morphology of L-PLGA and PGACL Wafers

**Fig. 3:**
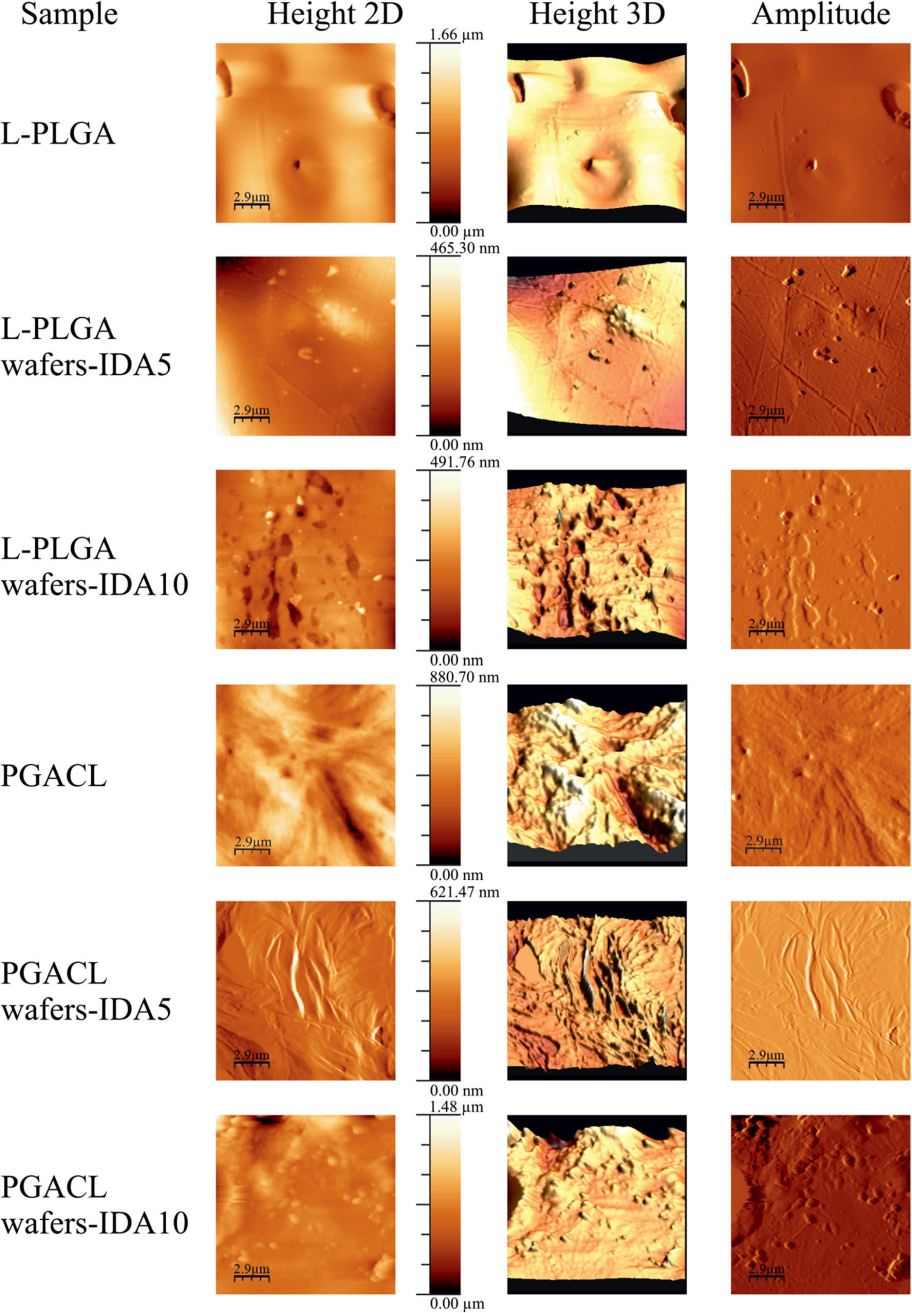
AFM images of “Height 2D”, “Height 3D” and “Amplitude” of L-PLGA wafers, L-PLGA wafers-IDA5, L-PLGA wafers-IDA10, PGACL wafers, PGACL wafers-IDA5 and PGACL wafers-IDA10.

The analysis of AFM images (Height 2D, Height 3D and Amplitude) for L-PLGA wafers showed a relatively monolithic surface with single pores. Slits and cracks were not observed. The incorporation of IDA into L-PLGA wafers resulted in the least differential topography. For L-PLGA wafers-IDA5, single granules were observed. Whereas the addition of 10% w/w of IDA influenced the presence of numerous immersions and hills (Fig. 3).

AMF images (Height 2D, Height 3D and Amplitude) for PGACL wafers showed a monolithic surface with numerous immersions and hills. The introduction of 5% w/w and 10% w/w of IDA into PGACL wafers did not significantly influence the morphological features (Fig. 3).

### IDA Release Process

**Fig. 4:**
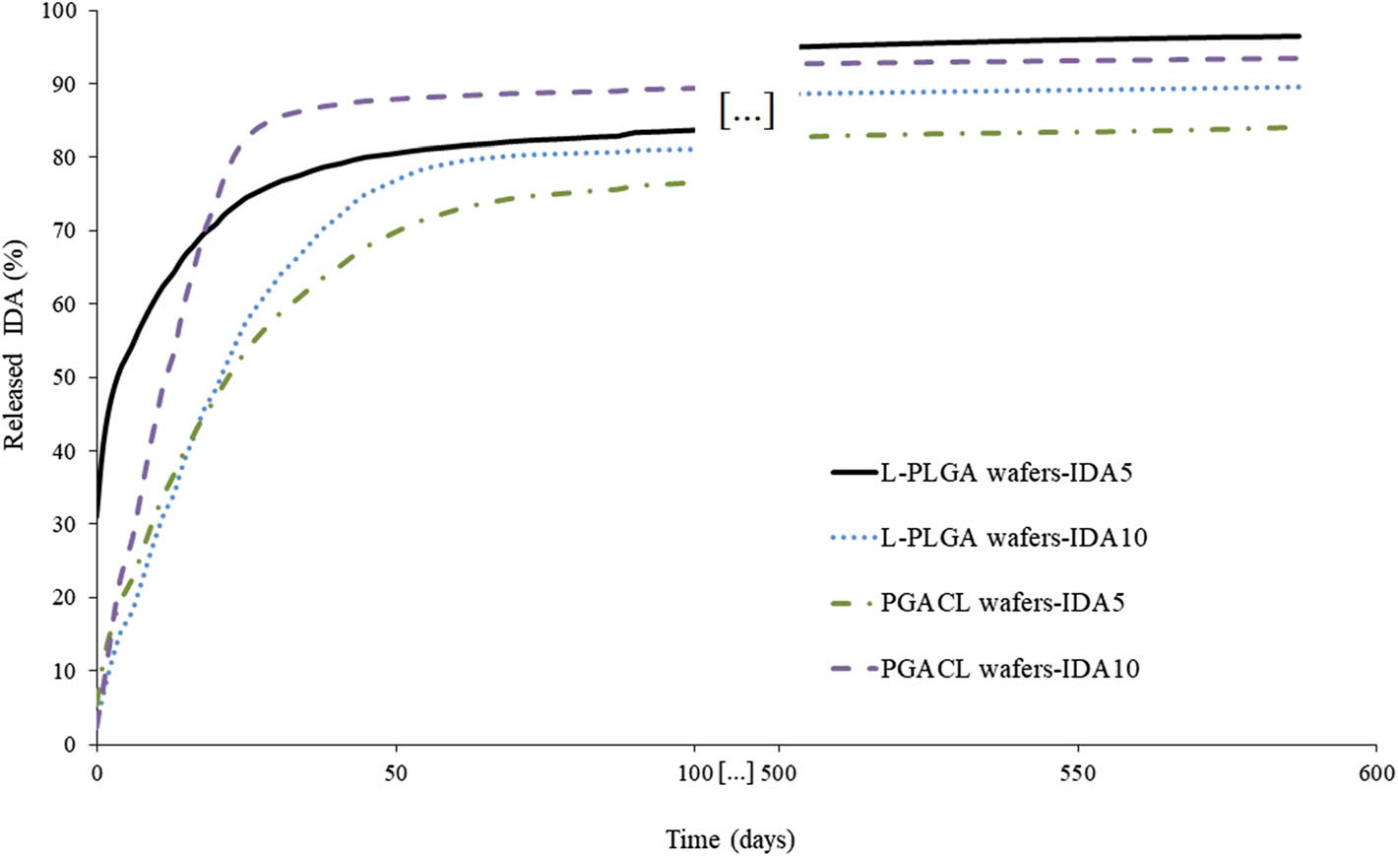
Cumulative profiles of IDA released from L-PLGA wafers-IDA5, L-PLGA wafers-IDA10, PGACL wafers-IDA5 and PGACL wafers-IDA10 incubated in aCFS over 587 days.

**Fig. 5:**
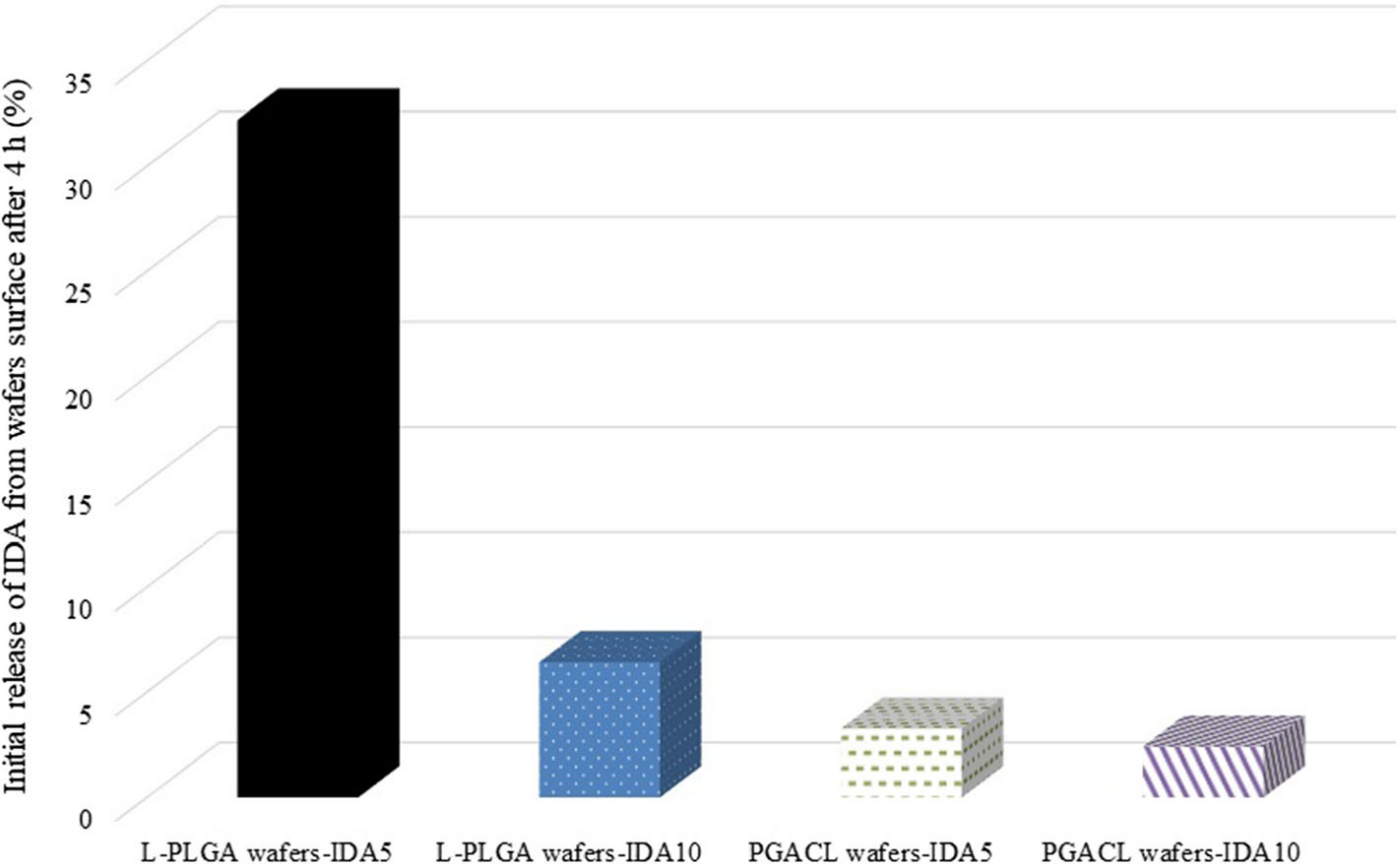
Initial release (%) of IDA from L-PLGA wafers-IDA5, L-PLGA wafers-IDA10, PGACLwafers-IDA5 and PGACL wafers-IDA10 from the surface after incubation for 4 h in aCFS.

IDA release from L-PLGA and PGACL wafers-IDA5 and wafer-IDA10 was performed over 587 days. After this period, the substance was not detectable. The analysis of the data representing the cumulative release of IDA showed similar profiles for all tested wafers. A bi-phasic model was observed for all kinds of wafers (Fig. 4). Differences in the initial release of IDA after 4 h were observed (Fig. 5). The largest amount of IDA was released from L-PLGA wafers-IDA5 (~32%), whereas for other wafers the release was significantly lower (Fig. 5). The profiles revealed a relatively fast release process over 30–60 days, indicating a rapid increase in IDA concentration. In the first phase, 85–91% of IDA was released, whereas in the second phase the remaining amount of IDA was released.

### IDA Release Kinetics

Comparison of the obtained IDA released in the first phase with mathematical models such as 0-order, I-order Higuchi and Korsmeyer–Peppas was performed for the first phase because 85–91% of IDA was released in this period.

The results showed a very good fit with all models for L-PLGA wafers-IDA10, PGACL wafers-IDA5 and wafer-IDA10 with R^2^ between 0.9182 and 0.9986 (Table II). L-PLGA wafers-IDA5 characterized the best fit for IDA release in the first phase with the Korsmeyer–Peppas model (R^2^ = 0.9865), whereas the worst was with 0-order kinetics (R^2^ = 0.6640). For the Korsmeyer–Peppas model, the diffusion exponent (n) was noted in the range from 0.51 to 0.53 (Table II).

**Table II:** Mathematical Matching of the IDA Release Curve in the First Phase with 0-order, I-order, Higuchi and Korsmeyer–Peppas Models

Wafer	L-PLGA wafers-IDA5	L-PLGA wafers-IDA10	PGACL wafers-IDA5	PGACL wafers-IDA10
Model	R^2^			
0-order	0.6640	0.9519	0.9182	0.9687
I-order	0.8870	0.9986	0.9800	0.9819
Higuchi	0.8563	0.9855	0.9944	0.9784
Korsmeyer – Peppas	0.9865 *n* = 0.51	0.9922 *n* = 0.53	0.9853 *n* = 0.52	0.9945 *n* = 0.52

n = diffusion exponent in Korsmeyer-Peppas model

### Degradation Process

The copolymer composition and chain microstructure were studied for the degradation of the PGACL wafer-IDA5 over 1255 days, with measurements performed every two weeks until the 126th day of degradation and then after the 1255th day of degradation.

**Fig. 6:**
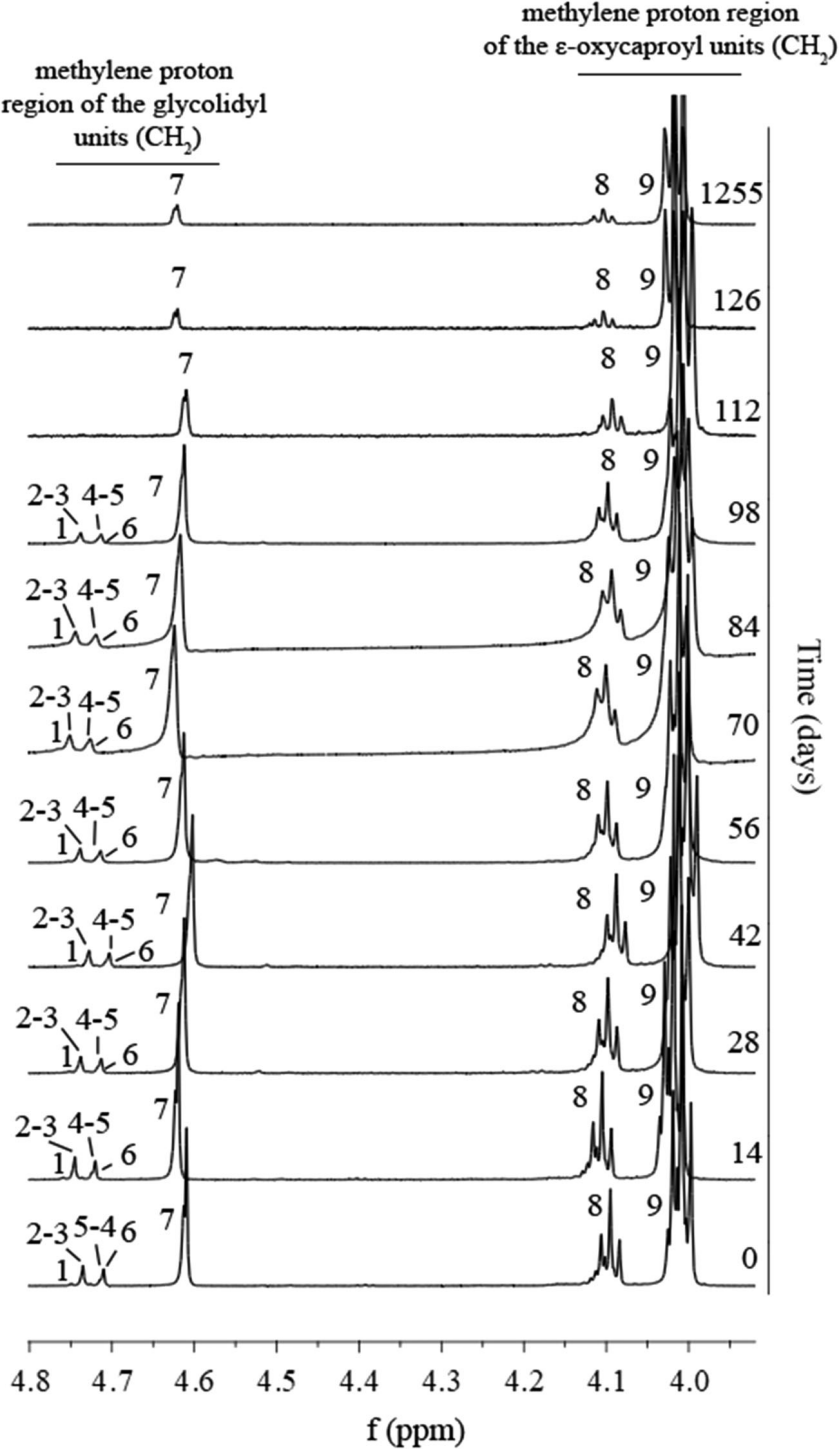
^1^H NMR spectra of PGACL wafers-IDA5 (600 MHz, DMSO-d6) after 0, 14, 28, 42, 56, 70, 84, 98, 112, 126 and 1255 days of degradation. Methylene proton region of the glycolidyl units (CH_2_): 1 – *GGGGG*, 2 – *CGGGG* + *GGGGC*, 3 – *CLGGGC*, 4 – *GGGGC*, 5 – *CGGGG* + *CGGGC*, 6 – *CGGC*, 7 – *CGC*. ε-methylene proton region of the ε-caproyl units (CH_2_): 8 – *CG*, 9 – *CC*.

Changes in the monomer unit distribution in the polymer chain of PGACL during the degradation process were determined on the basis of the ^1^H NMR spectra. Results obtained for the PGACL wafer-IDA5 before the degradation process revealed a low initial content of *F*_*GG*_ (⁓ 10 mol%) and a predominant content of *F*_*CL*_ (⁓ 90 mol%). During the 126-day period no significant changes in the content were observed, whereas after 1255 days of degradation a decrease of *F*_*GG*_ from 9.8 to 3.6 mol% was noted with a simultaneous increase of *F*_*CL*_ from 90.2 to 96.4 mol% (Fig. 6, Table III).

**Table III:** Changes in the Composition and Chain Microstructure for PGACL Wafers-IDA5 Over 1255 days of Degradation

Time (days)	0	14	28	42	56	70	84	98	112	126	1255
*F*_*GG*_	9.8	9.5	8.3	9.4	9.1	7.9	7.3	8.0	8.3	9.4	3.6
*F*_*CL*_	90.2	90.5	91.7	90.6	90.9	92.1	92.7	92.0	91.7	90.6	96.4
*l*_*GG*_	0.6	0.6	0.6	0.6	0.6	0.6	0.5	0.5	0.5	0.6	0.5
*l*_*CL*_	5.4	5.4	6.2	5.5	5.6	6.5	6.9	6.3	5.9	5.4	13.4
*R*	1.05	1.06	1.5	1.06	1.07	1.05	1.06	1.07	1.10	1.08	1.09

*F*_*GG*_ and *F*_*CL*_ – molar percentage of glycolidyl units and ε-caproyl units, respectively; *l*_*GG*_ and *l*_*CL*_–average length of glycolidyl blocks and ε-caproyl blocks, respectively; *R* – randomization ratio

Changes in the chain microstructure of PGACL were also analyzed on the basis of the ^1^H NMR spectra. The study of the PGACL wafer-IDA5 showed that an initial *l*_*GG*_ 0.6, *l*_*CL*_ 5.4 and *R* 1.05. No significant changes during the 126 days of degradation in the chain microstructure were revealed, whereas after 1255 days, *l*_*GG*_ did not show significant changes and substantial elongation for *l*_*CL*_ from 5.4 to 13.4 took place. No noticeable changes in the value of *R* during the degradation process were observed (Fig. 6, Table III).

### Idarubicin Decreases the Viability of U87MG Cells

**Fig. 7:**
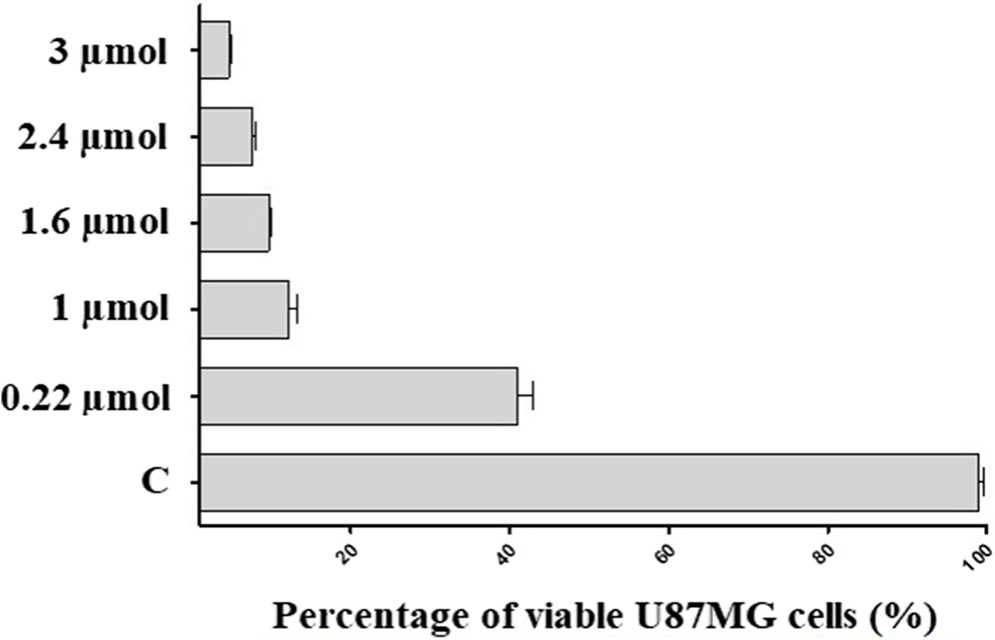
Influence of IDA on the viability of U87MG cells. C – untreated cells. The concentrations: 0.22 μmol, 1 μmol, 1.6 μmol, 2.4 μmol and 3 μmol of IDA reflect the cumulative amounts released from PGACL wafers-IDA5 over 4 h, 7, 14, 30 and 60 days, respectively.

The influence of IDA on the proliferation and viability of U87MG cells was determined by blue staining. The results were presented as a percentage of the viability of control cells (arbitrarily assigned 100% viability). Exposition of U87MG cells to IDA led to a significant reduction in cell viability (Fig. 7). The half maximal inhibitory concentration was revealed at a level of 0.15 μmol.

## Discussion

### Release Process of IDA

A bi-phasic model for IDA release for 587 days from all formulated wafers was revealed (Fig. 4), which is characteristic for PLGA small particles and PLGA thin films (31).

The solution casting method allowed the formulation of relatively thin wafers with IDA. Usually, the first phase is determined as a burst effect and is attributed to the release of non-entrapped substance molecules in the polymer structure or substance molecules close to the surface (32). Previously, the burst effect was determined as a phase of the release mechanism and as a surface phenomenon with a negative or positive role in the release process. Generally, a negative role is related with uncontrolled release which may influence the decrease in the total drug dose tailored for the formulation with prolonged release (33). Moreover, a toxic effect may take place. However, in the treatment of GBM by IDA, a positive aspect of the burst effect can be taken into account. The drug substance released during the burst effect may be recognized as the initial dose, which may be favorable from the point of view of a rapid invasion and of glioma relapse. In this study, in the first phase significant amounts of released IDA were observed. However, in the case of L-PLGA wafers-IDA5, the release of 31.05% of IDA was noted in the first four hours (Fig. 5). Simultaneously, in the second phase the smallest amount of IDA was released (Fig. 4). Therefore, other wafers (L-PLGA wafers-IDA10, PGACL wafers-IDA5 and PGACL wafers-IDA10) should be considered in further research because of the lower burst effect. However, for Gliadel® carmustine release at a level of ∼60% in three days was revealed (20). For that reason, further examination is needed.

It should be pointed out that in the case of Gliadel®, a maximum of eight wafers per surgical procedure may be administrated. The solutions proposed in this study for L-PLGA wafers-IDA10, PGACL wafers-IDA5 and PGACL wafers-IDA10 allow the application of various wafers, such as those with a fast first phase of release and a slower second phase of release to ensure a bolus dose and a maintenance dose in different clinical cases. Moreover, release was noted over ∼18 months (587 days), which may limit a relapse. The release period of IDA from L-PLGA and PGACL was optimized to the present median survival time of patients with GBM ( 18 months) (16). However, this suggestion requires further research. The release rate and dose may be regulated by the amount and kind of wafer for various therapeutic effects. The most universal wafers are PGACL wafers-IDA5 because these have the lowest amount of released IDA in the first phase and a stable and steady degradation (Fig. 6, Table III). Moreover, for this wafer, inhibition of proliferation of the U87MG cell line was shown. The half maximal inhibitory concentration for IDA was lower than the released doses from the wafer (Fig. 7), which may suggest great potential for the intracranial treatment of GBM.

Simultaneous high matching with various kinetic models may be evidence of complex degradation. For the Korsmeyer–Peppas model, n was in the range from 0.51 to 0.53 for the analyzed wafers, at which point a combination of both diffusion of the drug through the polymer and dissolution of the polymer take place (Table II).

### IDA-Copolymer Interactions

Polymer–drug substance interaction is one of the features determining the rate-controlling release mechanism in each phase of the release process. The analysis of the spectrum for the L-PLGA wafer-IDA10 revealed the lack of an additional band in comparison to the spectra of the L-PLGA wafer and pure IDA, which indicated if any bonds between IDA and L-PLGA had occurred (Fig. 2). Moreover, the spectrum obtained by subtracting the PGACL spectrum from the IDA-PGACL spectrum revealed almost the same spectrum as that of solid IDA (Fig. 2c). It also proved that in the PGACL wafer with IDA, intra- and intermolecular bonds were not preserved. Therefore, the prolonged release process of IDA from L-PLGA wafers-IDA5 and L-PLGA wafers-IDA10 resulted not from interactions between the drug substance and the polymer, but from the trapping of IDA. This fact also influenced a bi-phasic release pattern without slowing down and deteriorations in delivery (Fig. 4), which may be important for ensuring a bolus dose and a maintenance dose.

These results reflected the previous study on the release of doxorubicin from PGACL. An interaction between doxorubicin and PGACL was not detected. However, the opposite effect was observed for L-PLGA wafers with doxorubicin, in which hydrogen bonds between the hydroxyl groups of doxorubicin and the carbonyl groups of the copolymer were detected. The interactions between doxorubicin and L-PLGA resulted in inhibition of the release process over ~50 days (34).

### Wafer Surfaces

In the case of implantable drug delivery systems based on PLGA copolymers, the surface morphology plays an important role, particularly in the first phase of the release process, usually determined as a burst effect (31,33). In this study, a two-phasic model was observed for IDA released from the wafers, in which the first phase was relatively long (30–60 days) (Figs. 4 and 5). There are some morphology features influencing the release ratio during the first phase, i.e. the presence of pores, slits, cracks and disintegration of the formulation. It should be noted that the presence of water-filled pores may facilitate the release process. Release by water-filled pores is one of the known ways for a drug substance to be released from a formulation based on PLGA copolymers. Theoretically, the presence of pores increases the release from the surface and accelerates the release rate. On the other hand, partially closed pores and closed pores may inhibit the release process (35). Slits, cracks and disintegration are the unfavorable features that may appear during formulation, which may result in uncontrolled release and negative effects (36,37). Previously, various kinds of topography of wafers and rods were revealed, i.e. porous or monolithic, with various degrees of differentiation (33,38–40).

In this study, the AFM images showed a non-porous and monolithic surface for different types of wafers with IDA (Fig. 3). Simultaneously, in the first phase of the release process the largest amount of IDA was released from L-PLGA wafers-IDA5 (Fig. 5). Features such as slits, cracks and disintegration, which may result in an uncontrolled burst effect, were not observed. It should be pointed out that drug molecules bonded close to the surface are easily accessible for hydration (37). The non-porous, monolithic and the least differential topography surface of the L-PLGA wafers-IDA5 may ensure a controlled burst effect (Figs. 3 and 5) by facilitating the washout of drug molecules.

### PGACL Wafer Degradation

On the basis of the previous study on doxorubicin release from L-PLGA and PGACL with doxorubicin (5% w/w and 10% w/w) (34) and the current research on IDA release from L-PLGA and PGACL wafers-IDA5 and wafers-IDA10 (Figs. 4 and 5), PGACL wafers-IDA5 were chosen for the degradation study because of the most optimal release profile (Figs. 4 and 5). These wafers (PGACL with 5% w/w of doxorubicin and PGACL wafers-IDA5) revealed a relatively low burst effect and the prolonged release of doxorubicin (34) and IDA (Figs. 4 and 5) respectively.

The character of the release process is dependent on the degradation ratio. In this study, no significant changes in the composition and chain microstructure were observed during 126 days of degradation for the PGACL wafer-IDA5 (Table III), which indicated a stable and steady degradation process with stable release. In this period, 91% of the IDA was released. PCL is a hydrophobic, crystalline and slowly biodegradable polymer (41). PGA possesses a less hydrophobic character in comparison to PCL and generally degrades faster. Therefore, during degradation of the copolymer based on ε-caprolactone (CL) and glycolide (GA), the relatively fast decrease of the content of GA should be noted. However, the observations during this study did not confirm this fact, which reflects the previous study. This effect resulted from the resistance of CLGACL sequences to hydrolytic processes (41,42). A stable and steady degradation process is also confirmed by insignificant changes in the chain microstructure. Only for *l*_*CL*_ were fluctuations of values in the range from 5.4 to 6.9 observed (Table III). This phenomenon may indicate that crystallization with various intensities took place during the degradation period (Fig. 6), which confirms the previous study (24,41).

The degradation period resulted in IDA remaining in the wafer structure and influenced a long and linear release in the second phase (Fig. 4).

Wafer degradation performed over 1255 days allowed estimation of the residence period of PGACL wafers-IDA5 in brain tissue. In this period, a decrease of 95% of the mass was noted, however, an increase of *F*_*CL*_ and *l*_*CL*_ was observed after this period (Fig. 6, Table III).

Initially, R for PGACL wafers-IDA5 was slightly above 1, i.e. the chain microstructure was random (23). Moreover, this parameter did not change significantly during the degradation process (Fig. 6, Table III). This may guarantee regular degradation and controlled release at each phase.

## Conclusion

The study on biodegradable L-PLGA and PGACL wafers for the prolonged and local release of IDA points to the following: (i) solution casting is a proper method for the formulation of wafers with IDA, (ii) the release profiles ensure a bolus dose and a maintenance dose, (iii) surface properties influence the controlled burst effect, and (v) insignificant changes in the composition and chain microstructure during incubation in aCFS for the PGACL wafer-IDA5 points to a stable and steady degradation process.

L-PLGA and PGACL wafers with IDA formulated by solution casting methods are an interesting proposition with great potential in the intracranial treatment of GBM. However, at this stage of study, PGACL wafer-IDA5 showed the most optimal features for practical applications.

## Abbreviations

aCFS: Artificial cerebrospinal fluid
AFM: Atomic force microscopy
CL: ε-caprolactone
*D*: Molecular weight distribution
DSC: Differential scanning calorimetry
*F*_*CL*_: Molar percentage of ε-caproyl units
*F*_*GG*_: Molar percentage of glycolidyl units
*F*_*LL*_: Molar percentage of lactidyl units
FTIR: Fourier-transform infrared spectroscopy
GA: Glycolide
GBM: Glioblastoma multiforme
IDA: Idarubicin
*l*_*CL*_: Average length of ε-caproyl blocks
*l*_*GG*_: Average length of glycolidyl blocks
*l*_*LL*_: Average length of lactidyl blocks
L-PLGA: Poly(L-lactide-co-glycolide)
*M*_*n*_: Number average molecular weight
n: Diffusion exponent in Korsmeyer-Peppas model
NMR: Nuclear magnetic resonance
PGACL: Poly(glycolide-co-ε-caprolactone*)*
PLGA: Poly(lactide-co-glycolide)
*R*: Randomization ratio
*T*_*g*_: Glass transition temperature
*T*_*m*_: Melting temperature
